# Multiple ectopic recurrent germ cell tumors after total pineal mature teratoma removal: A case report and literature review

**DOI:** 10.3389/fonc.2023.1094231

**Published:** 2023-02-02

**Authors:** Lei Han, Ye Song, Luxiong Fang, Songtao Qi

**Affiliations:** Department of Neurosurgery, Institute of Brain Diseases, Nanfang Hospital of Southern Medical University, Guangzhou, China

**Keywords:** intracranial germ cell tumor, mature teratoma, tumor recurrence, pathogenesis, therapeutic strategy, case report

## Abstract

Intracranial germ cell tumors (GCTs) are highly heterogeneous and rare, and the recurrence of mature teratomas is uncommon. There is limited data on the systematic management of multiple recurrent tumors following total teratoma removal. Herein, we report repeated relapsing GCTs with different histological subtypes and locations after en bloc total resection of a pineal mature teratoma. A 14-year-old patient underwent total resection of a tumor in the pineal region and histopathology revealed a mature cystic teratoma. Four years later, the patient experienced a recurrence of the suprasellar tumor, which occurred several times over the next eight years. The tumor was successfully eliminated after multiple surgeries, radiotherapy and chemotherapy. By the time the paper was submitted, the patient had not had a recurrence of the tumor and was in the good physical condition and leading a normal life. Based on this case, we discussed the pathogenesis of recurrent mature teratoma and the therapeutic strategy of multiple recurrent GCTs.

## Background

Primary central nervous system (CNS) germ cell tumors (GCTs) are rare tumors of the nervous system that mainly occur in children and adolescents, accounting for approximately 3%–11% of all pediatric CNS tumors (1). They are highly heterogeneous with complex components. The latest WHO classification criteria for central nervous system tumors (2021) classified them as germinoma, mature teratoma, immature teratoma, teratoma with somatic-type malignancy, embryonal carcinoma, yolk sac tumor, choriocarcinoma and mixed GCT (2). All other subtypes except for germinoma were called non-germinomatous GCTs (NGGCTs). Although GCTs are curable tumors with multimodal therapy (3), roughly 10% of germinoma and 30% of NGGCTs recurred within 5 years (4, 5). The long-term outcome of recurrent patients with both germinoma and NGGCTs is not very optimistic. Murray MJ et al. reported a 55% 5-year survival rate for recurrent germ cell tumors and a 9% 5-year survival rate for NGGCTs with/without high-dose chemotherapy (6). As a type of NGGCTs, mature teratoma accounts for only 0.4% - 0.56% of intracranial GCTs and has a favorable prognosis with a 5-year survival rate of 88-100% treated by surgical resection alone (7).

Here, we describe a case with multiple ectopic recurrences after en bloc total resection of mature teratoma in the pineal region. Each recurrent tumor in this patient was located at a different site and accompanied by changes in histological subtypes. We discussed the management of these patients with multiple relapsing GCTs. The treatment timeline and tumor response, in this case, are shown in **Table 1**.

**Table 1:** Treatment timeline and tumor response in this case.

Disease	Year	Age	Tumor location	Tumor markers	Pathological diagnosis	Treatment	Tumor response
Initial tumor	2008	14	Pineal region	β-HCG 6.92 IU/L AFP was normal	Mature teratoma	S	CR
The 1^st^ recurrence	2012	18	Suprasellar region	β-HCG 1129 IU/L AFP was normal	Immature teratoma	C, S, C and R	After 2 courses of chemotherapy, the tumor did not shrink , and the tumor markers decreased to the normal range. After surgical resection, the patient received 4 courses of chemotherapy and radiotherapy, and finally achieved CR.
The 2^nd^ recurrence	2015	21	spinal cord in the lumbar spinal canal	β-HCG was normal AFP was normal	Germinoma	S and R	CR
The 3^rd^ recurrence	2017	23	Monro foramina and the fourth ventricle	β-HCG was normal AFP was normal	Germinoma	S, C, and ASCT+HDC	The tumor of the fourth ventricle was surgically removed, and the tumor of Monro foramina achieved PR after 5 courses of chemotherapy. After 2 ASCT+HDC, the patient finally achieved CR.

S, Surgery; C, chemotherapy; R, radiotherapy; ASCT, Autologous stem cell transplantation; HDC, High-dose chemotherapy; CR, Complete remission; PR, Partial remission.

## Case presentation

**Figure 1:**
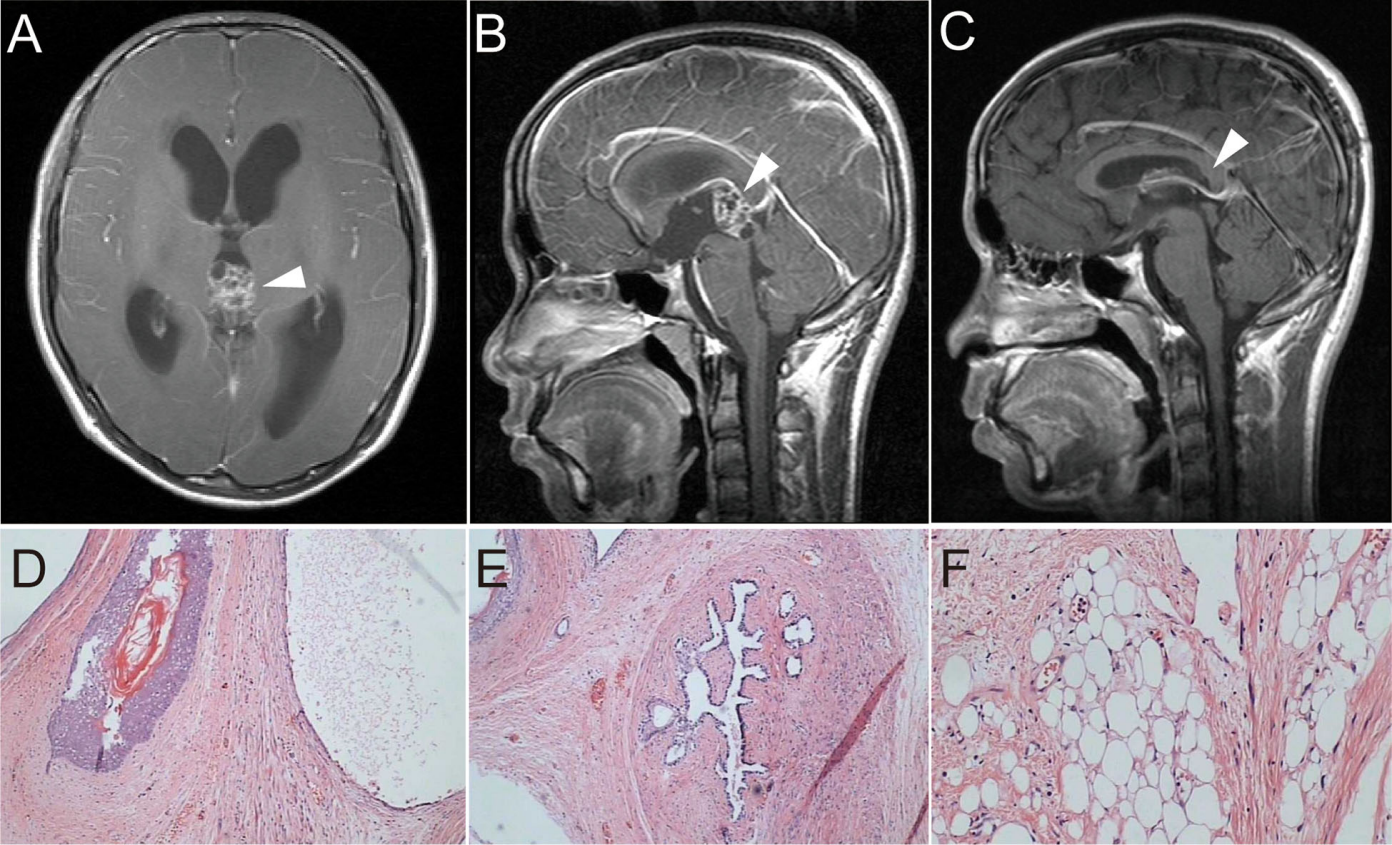
**(A, B)** T1-weighted imaging with contrast-enhanced MRI showed a pineal space-occupying lesion of heterogeneous enhancement and obstructive hydrocephalus. **(C)** Postoperative T1-weighted imaging showed no residual tumor or recurrence and reduced hydrocephalus. **(D–F)** Postoperative H&E) staining of tumor tissue showed hair follicles, breast duct-like structures, and adipose tissue (×100).

A 14-year-old boy with diplopia, headache, nausea, and vomiting was admitted to the neurosurgery department of our hospital in April 2008. Brain magnetic resonance imaging (MRI) suggested a pineal region mass and obstructive hydrocephalus (**Figures 1A, B**). Serum human beta subunit chorionic gonadotropin (β-HCG) was 6.92 IU/L and alpha-fetoprotein (AFP) levels were within the normal range. He then underwent surgery to remove the tumor completely, and hydrocephalus was relieved (**Figure 1C**). A histological examination of the tumor revealed a mature cystic teratoma (**Figures 1D–F**). He had an uneventful recovery and was discharged without further adjuvant therapy after the operation. A regular MRI follow-up was performed every 6 months. In June 2010, the MRI showed no sign of a recurrence, and serum tumor markers were within normal range.

**Figure 2:**
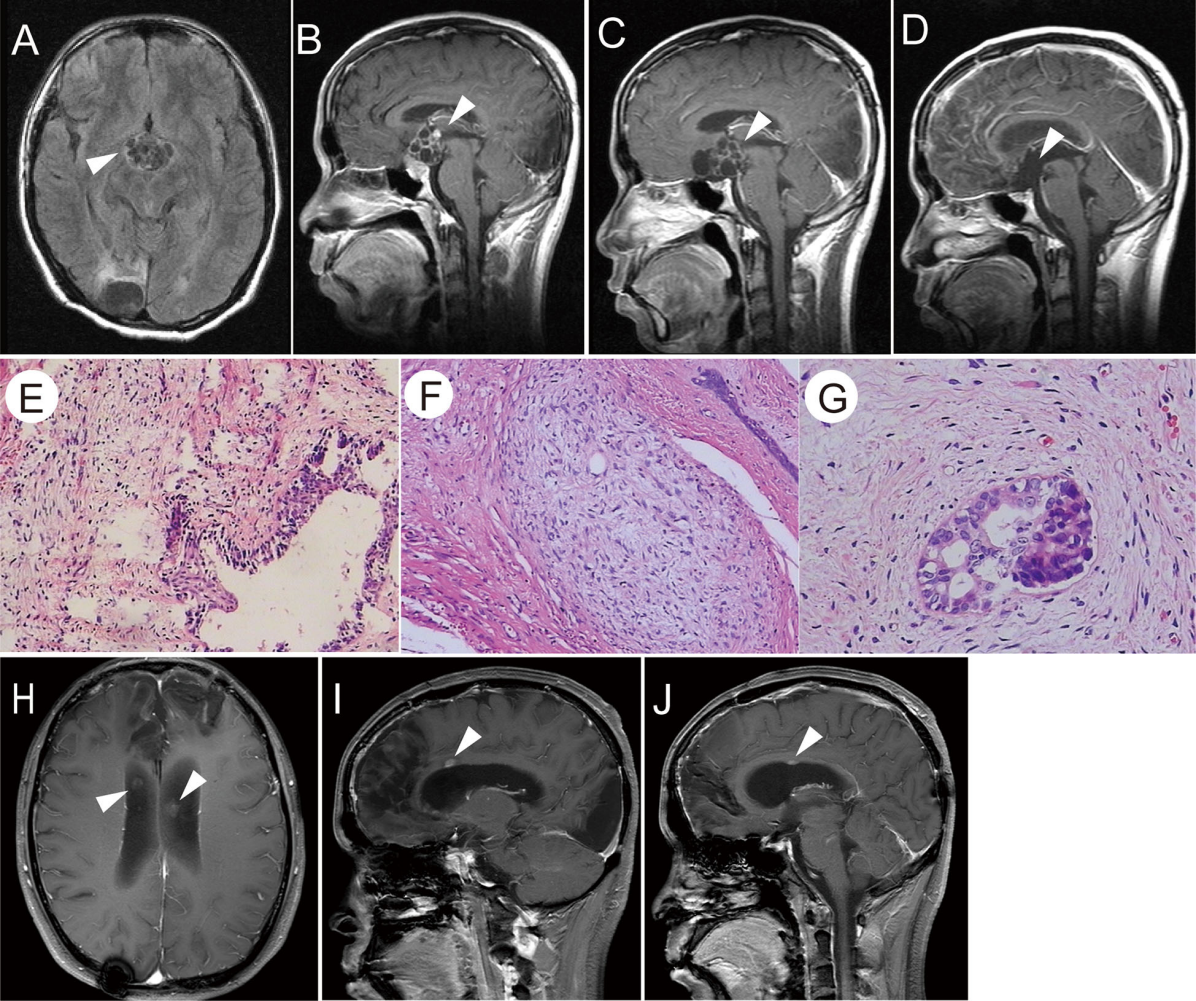
**(A, B)** T1-weighted images showed a suprasellar cystic solid tumor with an enhanced capsular wall and nodular shadow. **(C)** After chemotherapy, the enhancement of the capsule wall was weakened and the enhanced nodule disappeared; however, the tumor size was not reduced. **(D)** T1-weighted images showed no residual tumor or recurrence after the total removal of the suprasellar teratoma. **(E–G)** H&E staining of the tumor tissue showed that the cyst wall-like tissue was lined by columnar epithelium, and fibrous tissue proliferation was observed at the periphery. A small number of immature components was observed (E×100, F×200, G×400). **(H–J)** Post-second surgery and chemotherapy, T1-weighted images showed abnormally enhanced nodules in the midst of the corpus callosum, as indicated by the arrow.

In February 2012, the patient was admitted to the hospital because of a diminution of vision, polyuria, and polydipsia. MRI showed a space-occupying lesion in the suprasellar region (**Figures 2A, B**) with multiple scattered metastatic lesions in the lateral ventricle. Serum β-HCG levels were markedly elevated (1,129 IU/L) and AFP was within normal range. He received two courses of chemotherapy with bleomycin (3 mg days 1, 8, 15), etoposide (100 mg/m², days 1–5), and cisplatin (20 mg/m², days 1–5) (BEP) regimen. Subsequently, MRI showed a focal decrease in enhancement and disappearance of scattered metastatic lesions in the lateral ventricle. The value of serum β-HCG decreased to 0.603 IU/L. However, no significant shrinkage of the tumor size was observed (**Figure 2C**). Subsequently, he underwent tumor resection in May 2012 (**Figure 2D**), and histological examination revealed an immature teratoma (**Figures 2E–G**). He then received four courses of chemotherapy with the BEP regimen. However, a follow-up MRI showed multiple ventricular nodules (**Figures 2H–J**), and tumor recurrence was considered in January 2013. Furthermore, an MRI of the whole spinal cord revealed no abnormal signal. Therefore, he underwent three-dimensional conformal radiotherapy targeting the whole ventricle and bilateral frontal lobes (40 Gy) and 56 Gy to the tumor bed followed by three times of intrathecal methotrexate (10 mg).

**Figure 3:**
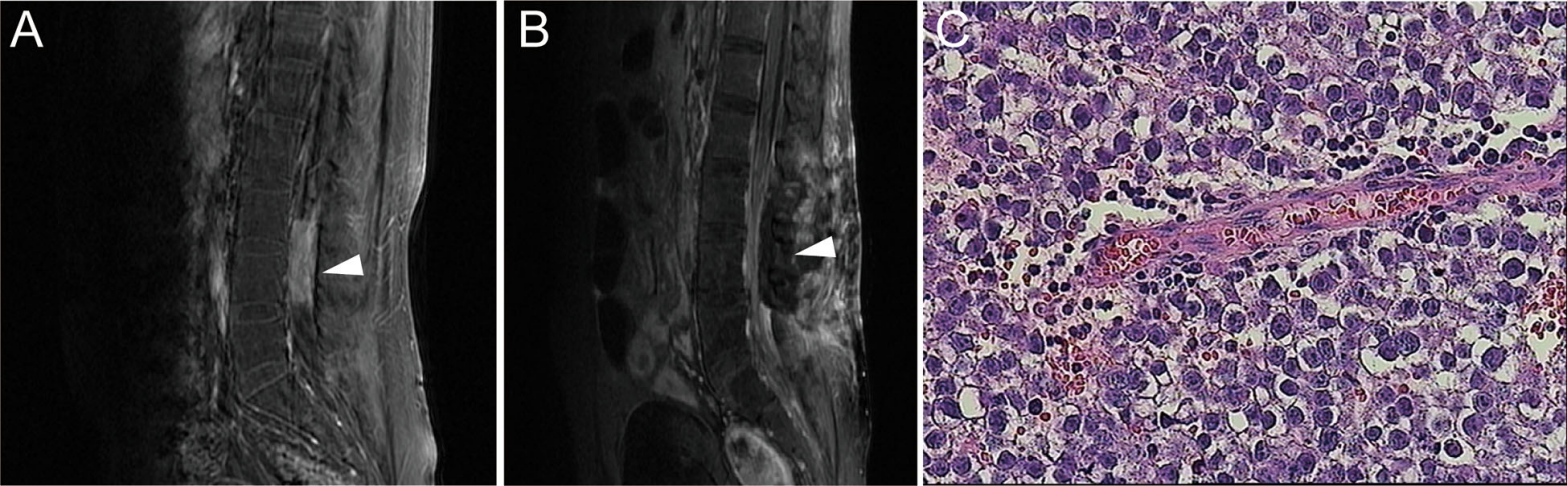
**(A)** Contrast-enhanced MRI showed a tumor with uniform density enhancement in the spinal cord of the lumbar spinal canal, L3-L4 disk levels (indicated by the arrow). **(B)** Postoperative MRI showed no residual tumor or recurrence. **(C)** H&E staining of the spinal tumor tissue (×400).

In May 2015, the patient had low back pain, and MRI revealed a space-occupying lesion of the spinal cord in the lumbar spinal canal, L3-L4 disk levels (**Figure 3A**). Serum β-HCG and AFP levels were within normal ranges and brain MRI indicated no sign of recurrence. Meanwhile, there was severe spinal cord compression, and the patient had the obvious symptom of pain. Therefore, he suffered from surgery to remove the mass (**Figure 3B**). Histological features of the tumor with large cells with transparent cytoplasm and small infiltrating lymphocytes indicated germinoma. (**Figure 3C**). The immunohistochemical staining was positive for placental alkaline phosphatase (PLAP) and c-kit (CD117), while HCG and AFP were negative. Subsequently, postoperative adjuvant radiotherapy (whole spinal irradiation) was administered.

**Figure 4:**
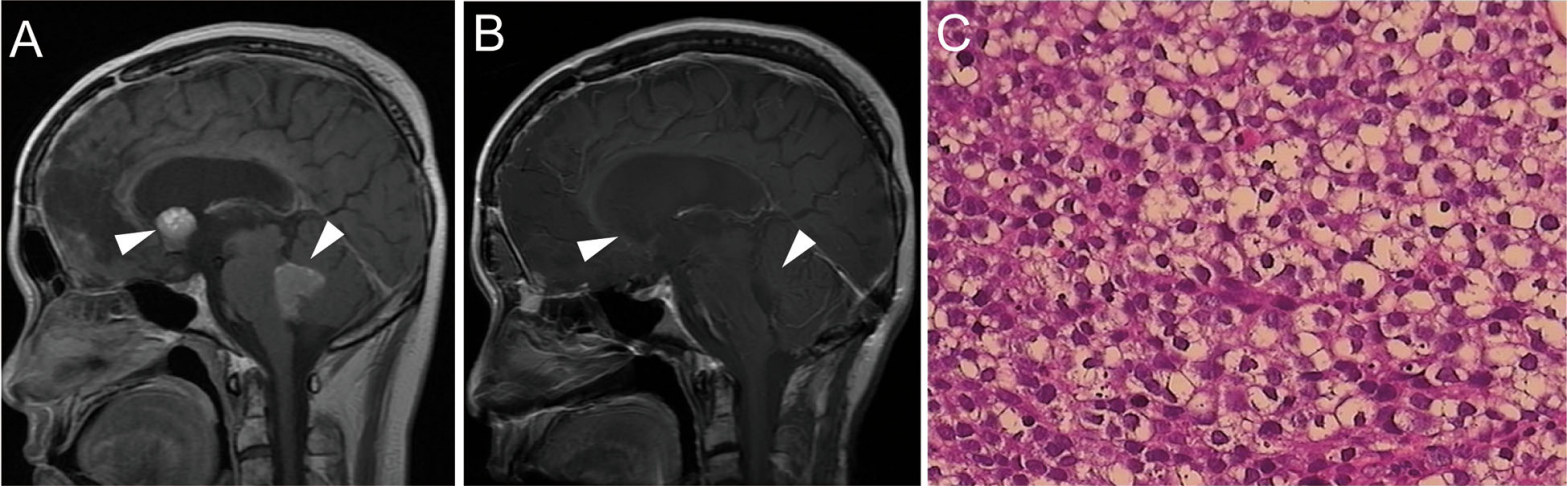
**(A)** Brain MRI revealed space-occupying lesions in the fourth ventricle and Monro foramina (indicated by the arrow). **(B)** After the operation and chemotherapy, tumors in both the fourth ventricle and Monro foramina disappeared. **(C)** H&E staining of the tumor tissue in the fourth ventricle (×400).

Regrettably, in March 2017, a follow-up brain MRI revealed two masses in the Monro foramina and the fourth ventricle (**Figure 4A**). Serum β-HCG and AFP levels were within normal ranges. Combined with the patient’s history and imaging examination, the lesions were more likely to be recurrence and metastasis of the teratoma or germinoma. The tumor in the fourth ventricle was relatively large, well-demarcated, and caused clinical symptoms, and the imaging showed signs of compression, accompanied by hydrocephalus. Therefore, we performed surgery to remove the tumor rather than stereotactic radiotherapy or dynamic observation. Histological features revealed germinoma (**Figure 4C**) and immunohistochemical staining was positive for PLAP and c-kit (CD117) and negative for HCG and AFP. After the operation, two courses of chemotherapy (etoposide 100 mg/m²: days 1–5) and cisplatin 20 mg/m²: days 1–5) were administrated, followed by three courses of etoposide (VP-16) (80 mg/m², days 1–5), ifosfamide (1500 mg/m², days 1–5) plus cisplatin (20 mg/m², days 1–5). Efficacy was evaluated as partial remission of the residual tumor in the Monro foramina during chemotherapy. Considering that the patient had severe bone marrow suppression many times during chemotherapy, high-dose chemotherapy supported by two rounds of autologous stem cell transplantation (ASCT) was administered. After two courses of high-dose chemotherapy, the tumor achieved complete remission (**Figure 4B**). By the time the paper was submitted, the patient had not had a recurrence of the tumor and was in good physical condition and leading a normal life. In terms of endocrine function, the patient still requires low-dose thyroxine and prednisone replacement therapy.

## Discussion

Intracranial mature teratomas are benign tumors with good prognoses, and the standard treatment is surgery (7, 8). Adjuvant therapy is generally unnecessary for mature teratomas after total removal due to resistance to chemotherapy and irradiation (7, 9). However, partial resection of mature teratoma may result in the rapid growth of a residual tumor that may require a second surgical resection (10). In addition, metachronous intracranial GCTs and late recurrent tumors have previously been reported after complete mature teratoma removal (7, 11, 12). Therefore, the treatment of intracranial mature teratoma should be total resection as much as possible, and long-term follow-up should be carried out after the operation.

The treatment of intracranial GCTs is closely related to the tissue components of the tumor. Germinomas are sensitive to radiotherapy and chemotherapy, and the overall 10-year survival rate is more than 90% (13). For NGGCTs, standardized radiotherapy and/or chemotherapy and appropriate surgery are important ways to improve survival. In recent years, with the development and maturity of neuroendoscopic technology, the treatment strategy based on pathological results of endoscopic biopsy has gradually been recognized. However, the positive rate of neuroendoscopic biopsy remains to be improved. Ahmed et al. (14)showed the false positive rate of endoscopic biopsy was 15%, and the proportion of secondary surgery or biopsy was 60%. Notably, the inconsistency rate between the second surgery and the initial biopsy was 21% in the 60% of patients. ​For pure germinomas, a biopsy is easy to confirm the diagnosis, but NGGCT components are complex, and restricted biopsy tissue is difficult to conclude the histological subtype. At the same time, for mature teratoma, even if the diagnosis is confirmed by biopsy, a second operation is required to remove the tumor. On the other hand, with the development of micro neurosurgery, the safety of craniotomy has been significantly improved. ​Qi S et al. (15) demonstrated that over 91% of patients with pineal region tumors can achieve a gross total resection, while surgical mortality is less than 1%. Consequently, for patients with highly suspected teratoma on imaging and tumor markers were not markedly evaluated, direct surgical resection of the tumor can be considered to decrease the risk of secondary surgery after biopsy.

Intracranial GCTs are complex in components and vary in sensitivity to radiotherapy and chemotherapy. Residual tumor of malignant intracranial GCTs after radiotherapy and/or chemotherapy is likely to be mature teratoma insensitive to radiotherapy and chemotherapy (16). Surgical resection of residual tumors is safe and effective for the residual tumors (8, 16). Ogiwara H et al. showed that secondary-look surgery should be encouraged relatively early after chemotherapy when tumor markers are normal or nearly normal but the size of the residual tumor increases or doesn’t change (17). We also believe that these types of tumors should be removed as early as possible so that they do not grow too large and increase the difficulty and risk of surgery. At the same time, it can also reduce the unfavorable effects of adverse reactions such as bone marrow suppression after chemotherapy on surgery.

In this case, the MRI features and near-normal tumor markers of the initial tumor are highly suspected teratoma. The tumor was resected *via* the occipital tentorial approach, and postoperative pathology verified mature teratoma. The patient avoided a second surgical removal of the tumor after biopsy confirmation. Four years after the total resection of mature teratoma of the pineal region, the patient developed a suprasellar tumor. The tumor was considered to be a GCT combined with a history of pineal teratoma and markedly elevated β-HCG. Due to the significant elevation of serum markers and the dissemination of ventricles, secondary hypothalamus injury may occur after suprasellar area surgery. Hence, the patient received BEP chemotherapy first. After two courses of chemotherapy of the BEP regimen, the disseminated lesions disappeared and the enhancement of the sellar tumor decreased. In addition, serum β-HCG also decreased to the normal range. Nevertheless, the size of the sellar region tumor did not shrink, even increased slightly, and the patient developed severe myelosuppression. This means that the tumor is insensitive to chemotherapy and needs to be removed surgically. Postoperative pathological features were consistent with immature teratoma. The tumor was considered as a mixed GCT or immature teratoma combined with the markers before chemotherapy. Subsequently, the patient developed recurrent germinomas of the lumbar spinal cord 3 years after the tumor removal and the fourth ventricle and Monro foramen 5 years after the surgery. Two recurrent tumors were successfully treated after multimodal therapy, including surgery, radiotherapy, and high-dose chemotherapy supported by ASCT.

Metachronous and late recurrent GCTs have been reported after the total removal of mature teratoma. Therapy strategies have also been reported for recurrent intracranial GCTs (6, 18). However, as far as we know, there are no serial studies on recurrent GCTs with multiple, different sites and different pathological subtypes. The case we reported had the following characteristics: 1. Ectopic recurrent tumor after en bloc total removal of mature teratoma; 2. The main focus of multiple recurrences was the site of no previous tumor; 3. Despite repeated tumor relapses, the patient achieved long-term survival. In theory, en bloc complete resection of the tumor without a spread of metastasis can remove all the tumor components contained in it, and recurrence of malignant components without adjuvant therapy at a four-year interval is very rare. However, given the slight elevation of serum β-HCG at the time of initial diagnosis, a small component of immature teratoma may be present. In addition, the last two recurrent tumors were pure germinomas. Previous studies have shown that mature teratomas frequently have germinoma component (9). Thus, the initial mature teratoma may contain small amounts of immature teratoma or germinoma components. The initial tumor tissue was reexamined histologically and immunohistochemically, but no immature teratoma or germinoma components were found. ​In addition, no lesions were found in the suprasellar region during the initial diagnosis of the brain MRI and 2 years of regular follow-up after the surgery. An MRI of the whole spinal cord taken at the time of the initial episode also revealed no abnormalities. These imaging findings suggest that the tumors in the supraseller and pineal regions did not occur simultaneously. Therefore, the suprasellar tumor is likely to be a metachronous tumor, which is also consistent with the theory of the multi-center origin of GCTs. Cases of new GCTs occurring long after total removal of mature teratomas have been similarly reported (19, 20). In these “recurrent tumors”, germinoma seems to be more common (19). Meanwhile, in almost all of these cases, only one recurrent tumor was reported, and both primary and recurrent sites were intracranial. Although this case also presented with a different location several years after the total removal of a mature teratoma, this case experienced more relapses. At the same time, the recurrence site is throughout the central nervous system including the intracranial and spinal cord. Interestingly, despite multiple recurrences, each time the primary lesion was located in a previously uninvolved location. Furthermore, in multiple recurrences, the germinoma component that was most sensitive to chemoradiotherapy appeared later and eventually becomes the only component. Therefore, compared with the previously reported cases, the case presented here has more recurrence times, involves a wider range, and has certain unique features. This case experienced GCTs from the intracranial to the spinal cord and underwent treatment protocols for both germinoma and NGGCTs. It is an educational case with some lessons for the understanding and management of multiple sites and multiple recurrences of GCTs after total mature teratoma removal.

We report a case of repeated ectopic GCTs after en-bloc total removal for mature pineal teratoma with changes in tumor sites and pathological subtypes. This case suggests the possibility of multiple recurrent tumors after total resection of mature teratoma. Patients with multiple relapses of GCTs can also achieve long-term survival with multimodal therapy based on histopathologic diagnosis, including reasonable surgery, standardized and high-dose chemotherapy, and radiotherapy.

## Data availability statement

The original contributions presented in the study are included in the article/supplementary material. Further inquiries can be directed to the corresponding author.

## Ethics statement

Written informed consent was obtained for the publication of this case report from the patient/participant’s guardian/next of kin.

## Author contributions

LH collected the data, searched the literature, and drafted the manuscript; STQ, LXF, and YS participated in the operation and management of the patient. YS and LXF prepared radiological and histology figures. STQ designed the study, reviewed the manuscript, and revised the manuscript. All authors contributed to the article and approved the submitted version.
